# Supplementary material for: Association between oral microbiome and seven types of cancers in East Asian population: a two-sample Mendelian randomization analysis
Source: Front Mol Biosci. 2023 Nov 21;10:1327893. doi: 10.3389/fmolb.2023.1327893 (PMC10702768; doi:10.3389/fmolb.2023.1327893)
Supplement: Supplementary file 1 [file DataSheet1.ZIP › Supplementary Materials/MR plots of saliva/saliva═╝/colorectal cancer/pheno.437_to_colorectal cancer_forest.pdf]

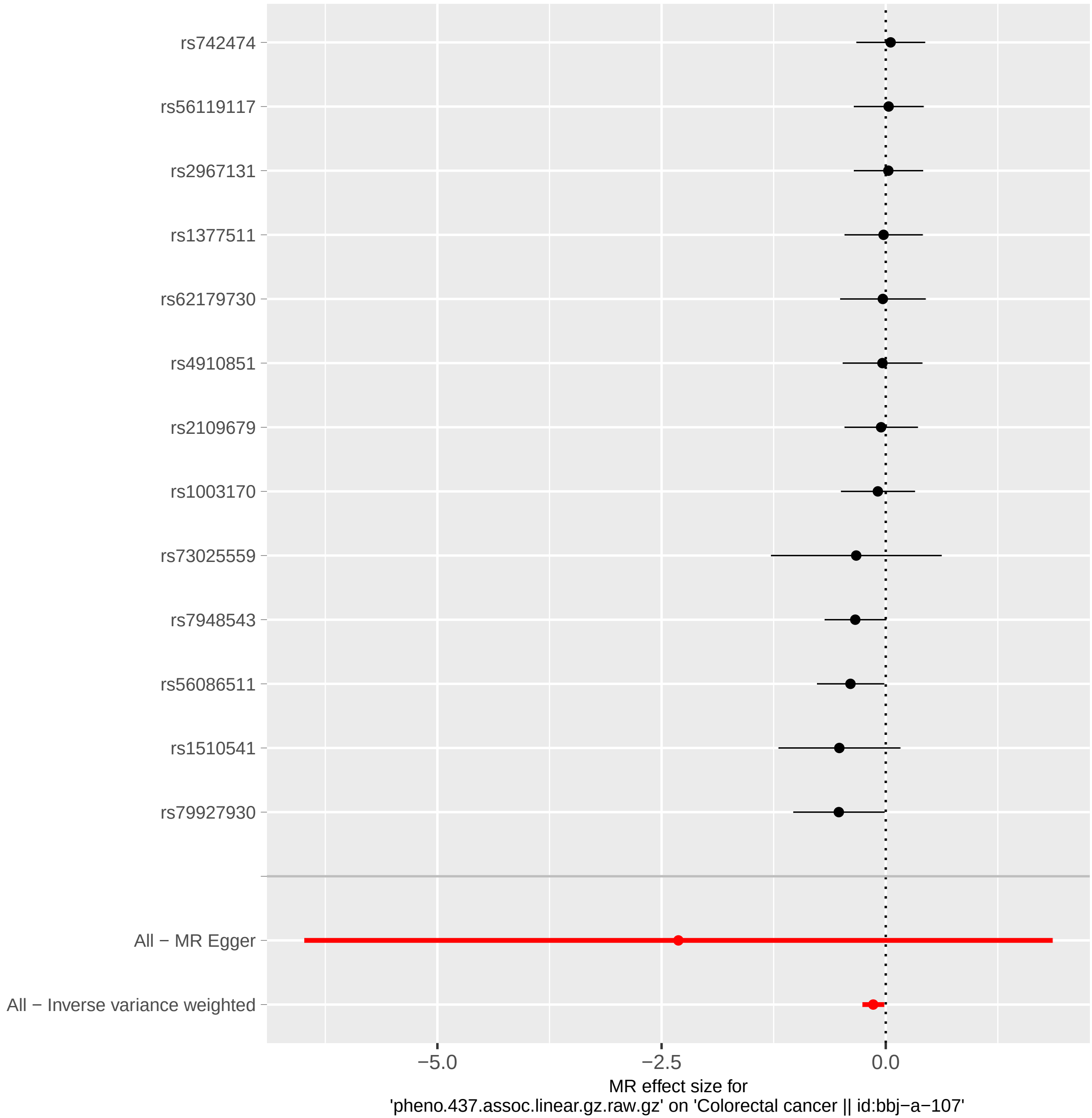

rs742474
rs56119117
rs2967131
rs1377511
rs62179730
rs4910851
rs2109679
rs1003170
rs73025559
rs7948543
rs56086511
rs1510541
rs79927930
All − MR Egger
All − Inverse variance weighted
−5.0
−2.5
0.0
MR effect size for
'pheno.437.assoc.linear.gz.raw.gz' on 'Colorectal cancer || id:bbj−a−107'
